# Clinico-Epidemiological Profile of Allergic Contact Dermatitis and Its Correlation With Patch Testing in a Tertiary Care Center in Eastern India

**DOI:** 10.7759/cureus.32119

**Published:** 2022-12-01

**Authors:** Satyajit Sahu, Mitanjali Sethy, Laxman Besra, Suvigya Sachan, Hemanta K Kar, Basanti K Devi

**Affiliations:** 1 Dermatology Venereology and Leprosy, Kalinga Institute of Medical Sciences, Bhubaneswar, IND

**Keywords:** ppd, acd, nickel, parthenium, patch test, allergic contact dermatitis

## Abstract

Background: Allergic contact dermatitis (ACD) is one of the most common skin disorders seen among patients attending dermatology clinics in India. Patch testing is the gold standard for diagnosing ACD. The clinical-epidemiological pattern of ACD and the allergen-causing it may be different in different geographic locations. Finding the profile of allergens commonly causing ACD in a particular region will help to formulate prevention strategies for the development of ACD.

Aim and objective: The primary aim of the study was to find out the clinical-epidemiological distribution of allergic contact dermatitis and to identify the common allergens causing it by patch testing in this region of India.

Materials and methods: A total of 111 cases of ACD were included in the study. Clinico epidemiological profiles of all patients were documented. The patch testing was performed in the outpatient department using the antigens of the Indian Standard Series kit (Systopic Laboratories Pvt. Ltd., New Delhi, India). Patches were removed after 48 hours (two days) of application. The first reading was taken 15 to 20 minutes after the removal of patches on day two. A second reading was taken on day four (96 hours of application) to confirm the presence of an allergic reaction.

Results: The patch test was found to be positive in 69% of cases. It was observed that male persons from lower socioeconomic status were getting ACD on most accounts. Potassium dichromate (PDC) was found to be the most common allergen (30.43%) followed by parthenium (26.08%), para-phenylenediamine (PPD) (21.73%), nickel sulfate (18.84%), chlorocresol (15.94%), black rubber (14.49%), cobalt sulfate (13.04%), and wool alcohols (7.24%) respectively.

Conclusion: Our study showed potassium dichromate is the commonest allergen causing ACD in this part of the country. The importance of patch testing lies mainly in educating the patient regarding the avoidance of exposure to particular allergens to avoid the development of new ACD as well as an exaggeration of pre-existing ACD.

## Introduction

Among multiple skin diseases found in India, allergic contact dermatitis (ACD) is one of the common skin problems found in patients attending the dermatology outpatient department. It is considered as the inflammation of the skin that clinically presents as varying degrees of erythema, edema, scaling, and/or vesiculation. It is a cell-mediated disease involving both the adaptive and innate immune systems and is the prototype of a delayed-type hypersensitivity reaction [1]. It occurs more commonly in adults and up to 20% of the adult population is sensitized to one or more contact allergens [1]. The diagnosis of ACD is relatively simple as it can be elucidated by clinical history and/or by a patch test.

Patch testing (PT) is the gold standard for diagnosing ACD. Many times, history and clinical examination alone are not adequate to evaluate a patient’s contact allergens fully. The procedure of patch testing is quite easy to perform especially with preloaded allergens. However, the interpretation of the patch test needs an appropriate level of experience to properly classify the results, testing the relevant allergens in their proper vehicle and concentration [2]. The pattern of ACD and the allergen-causing ACD is quite different in different geographic locations. Finding the profile of allergens commonly causing ACD in a particular region will help in formulating prevention strategies for the development of ACD. The present study was carried out with the objectives to study the clinico-epidemiological distribution of ACD and identify the common allergens causing it by using a patch test.

## Materials and methods

This was a prospective observational study carried out in the department of dermatology in a tertiary care center in eastern India. A total of 111 cases of ACD were included in the study. Eleven patients were lost to follow-up at different points in time. Finally, a sample of 100 patients’ data was analyzed. Before the commencement of the study, approval was obtained from the institutional ethics committee, Kalinga Institute of Medical Sciences, Kalinga Institute of Industrial Technology (KIIT) University, Bhubaneswar, Odisha, India (approval no.: KIMS/KIIT/IEC/73/2017). Patients clinically diagnosed to have ACD, willing to participate in the study, and agreeing to undergo patch testing were included in the study. Patients with any other pre-existing skin disorders, on immune suppressive therapy, pregnant women, lactating mothers, and patients who refused patch testing were excluded from the study.

Interpretation

Interpretation of patch testing results was done as described in Table 1 [3].

**Table 1:** Interpretation of patch test result

Grading	Clinical finding	Evaluation
+ or ?	Faint erythema	Doubtful reaction
+	Erythema, infiltration, and/or discrete papules	Weak positive reaction (non-vesicular)
++	Erythema, infiltration, papules, and vesicles	Strong positive reaction (vesicular)
+++	Intense Erythema, infiltration, and coalescing vesicles	Extreme positive reaction (bullous)

Methodology

All patients clinically suspected to have ACD were included in this study. A thorough history was documented with particular reference to duration, onset, the evolution of the symptoms, systemic disturbances, any pre-existing skin diseases, seasonal variation of the disease, site of involvement, the morphology of the lesion, distribution of lesion, and occupation of the patient. Any personal and/or family history of atopy was also noted down. Past history of similar symptoms was documented. Blood investigations such as routine hemograms and fasting blood sugar were advised whenever necessary. Based on the type of exposure to allergens, the patients were patch tested with the appropriate antigens. The necessity and importance of the patch test were explained to the patients, and informed consent was taken. The patch testing was performed in the outpatient department using the antigens of the Indian Standard Series kit (Systopic Laboratories Pvt. Ltd., New Delhi, India), approved by the Contact and Occupational Dermatoses Forum of India (CODFI). The allergens included are listed in Table 2.

**Table 2:** List of allergens used in the patch test

SI. No	Name	Concentration in %
1	Petrolatum	100%
2	Wool alcohols	30%
3	Balsam of Peru	10%
4	Mercaptobenzothiazole	1%
5	Potassium dichromate	0.1%
6	Nickel sulfate	5%
7	Cobalt sulfate	5%
8	Colophony	10%
9	Epoxy resin	1%
10	Paraben mix	9%
11	Para-phenylenediamine	1%
12	Parthenium	15%
13	Neomycin sulfate	20%
14	Benzocaine	5%
15	Chlorocresol	1%
16	Formaldehyde	2%
17	Fragrance mix	8%
18	Thiuram mix	1%
19	Nitrofurazone	1%
20	Black rubber mix	0.6%

Before the application of antigens, the upper portion of the back of the patient was gently cleaned with sterile gauze. The patch test unit was marked with waterproof ink with the names of the antigens to be tested. Fifteen to 100 microliters of allergens were incorporated in petrolatum, which was subsequently introduced in chambers or cups with a diameter of 10 mm to 15 mm, which were made up of aluminum foil. Allergens were applied on the patch test unit with the first allergen in the top right-hand corner and then downwards in the region of the upper back. The control was applied on the left side of the vertebral column in parallel to the allergens on the right side. Patients were advised to keep the patch test in place for 48 hours, avoid taking baths or wetting the back, rubbing and lying on the back, exercising, or any other activity causing sweating, tight garments, scratching the patch test site, exposure to sunlight/UV light, and to report immediately if there is severe itching or irritation, and to come after 48 hours and 96 hours for patch test reading. Patches were removed after 48 hours (two days) of application. The first reading was taken 15 to 20 minutes after the removal of patches. A second reading was taken on day four (96 hours of application) to confirm the presence of an allergic reaction.

## Results

In our study, the majority of the patients (20%) were in the age group of 41 to 50 years followed by 51 to 60 years and 31 to 40 years which accounted for 19 % each. The mean age of the patients was 42.8 years. This study revealed ACD is more common in males (55%) than females (45%) with a male-to-female ratio of 1.22:1. Majority of patients (50%) belonged to the lower socio-economic status (LSES) group and most of the patients were from urban areas (72%) than rural areas (28%).

The average time lag between exposure to allergen and development of ACD was found to be two to five years. The most common presenting complaint was itching i.e., 96% followed by scaling in 58% of cases, which were associated with other complaints like redness, swelling, pain, burning, etc. The commonest site of involvement was the hand i.e., 26% followed by the head and neck, which accounted for 24% of cases. Around 32% of cases complained of multiple site involvement, primarily affecting the hands. Most of the patients (32%) presented to us having a duration of complaint to be of two to six months. Around 25% of the study participants presented with complaints for about a week to one month. Association of atopy was detected in 18% of cases. The nail was involved in 30% of cases, of which nail discoloration was the most common sign reported in 23% of cases followed by pitting and paronychia.

Upon patch testing, 69% of the patients showed one or more positive patch test results (Table 3).

**Table 3:** Distribution of cases as per the positive reaction to allergens (n=69)

Allergen	Frequency in numbers	Frequency in %
Wool alcohols	5	7.24
Formaldehyde	4	5.79
Mercaptobenzothiazole	1	1.44
Potassium bichromate	21	30.43
Nickel sulfate	13	18.84
Cobalt sulfate	9	13.04
Colophony	4	5.79
Epoxy resin	3	4.34
Paraben mix	4	5.79
Para-phenylenediamine	15	21.73
Parthenium	18	26.08
Neomycin sulfate	2	2.89
Benzocaine	4	5.79
Chlorocresol	11	15.94
Fragrance mix	4	5.79
Thiuram mix	3	4.34
Nitrofurazone	3	4.34
Black rubber mix	10	14.49
Multiple allergens	36	52.17

It was found that ‘1+’ is the most common (42%) intensity of the reaction in the patch test followed by ‘faint reaction’ detected in around 22% of cases while ‘negative reaction’ was reported in 31% of the cases (Table 4).

**Table 4:** Distribution of study participants according to the intensity of reaction to the patch test

Reading of patch test	Frequency in %
Negative	31
Faint reaction	22
1+	42
2+	5
3+	0
Irritant reaction	0
Not tested	0

**Figure 1:**
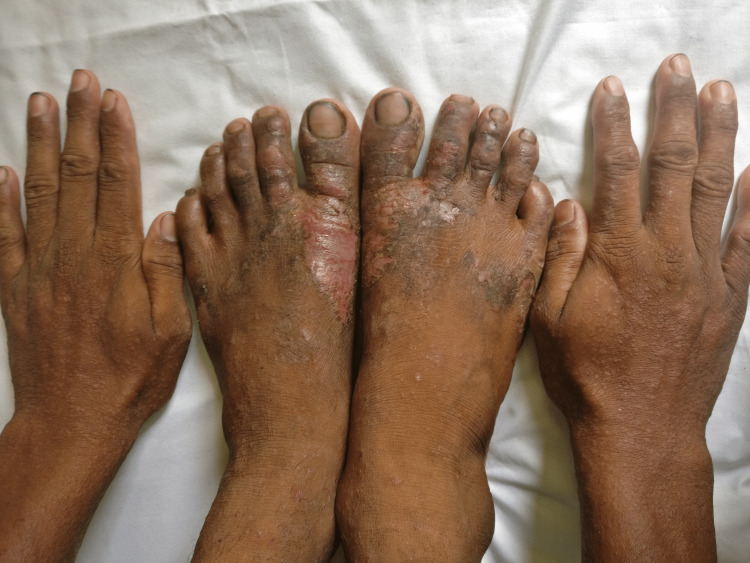
Allergic contact dermatitis due to cement

**Figure 2:**
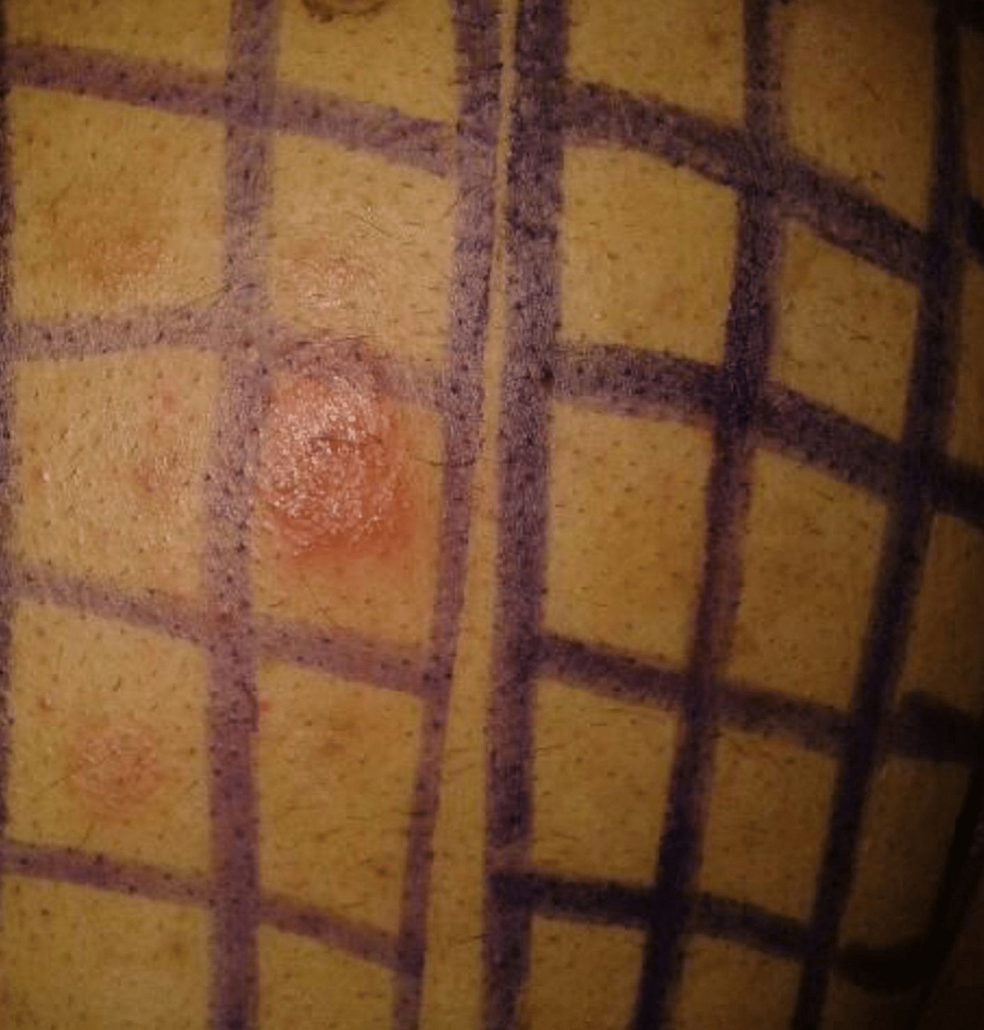
Patch test showing positivity to parthenium

**Figure 3:**
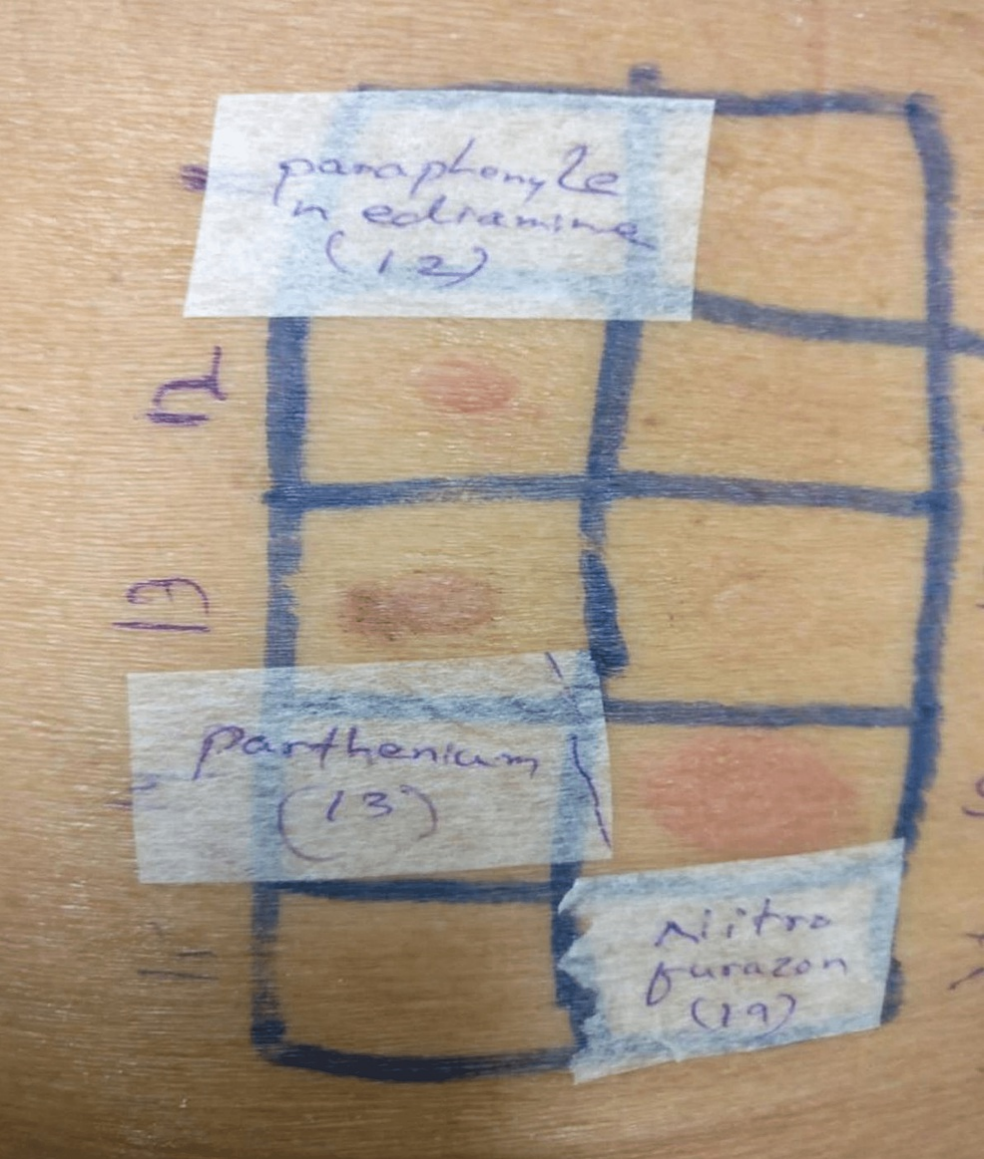
Patch test showing positivity to PPD, parthenium, and nitrofurazone PPD: Para-phenylenediamine

Cement was found to be the most common cause of ACD in the present study (30.43%) (Figure 1). The most common allergen in cement is hexavalent chromium, and the sensitivity to it was demonstrated by a closed patch test with 0.1% potassium dichromate. Parthenium was the second most common allergen causing ACD in our study participants. Around 26.08% of cases showed positive patch test reaction to parthenium (Figure 2). Para-phenylenediamine occupied the third place, involving 21.73% of patients (Figure 3).

**Figure 4:**
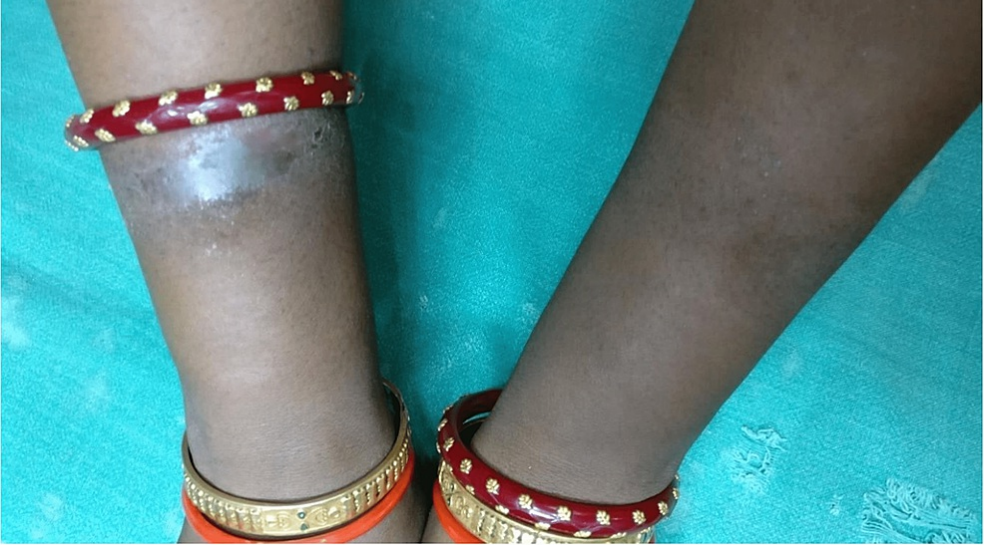
Allergic contact dermatitis due to artificial jewelry (nickel)

**Figure 5:**
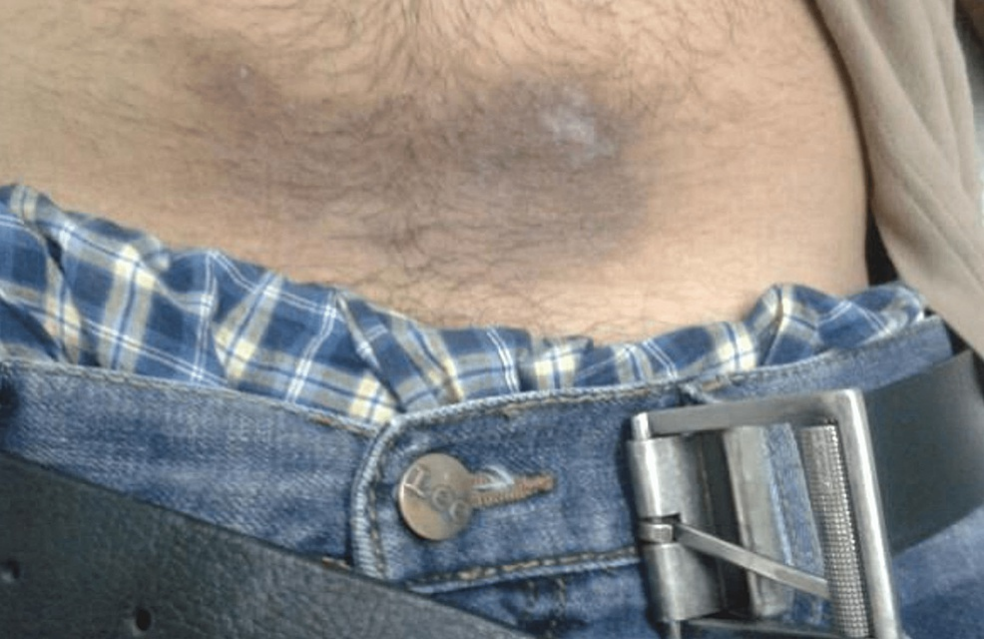
Allergic contact dermatitis due to belt buckle (nickel)

In our study, ACD to nickel was confirmed in 18.84% of patients (Figures 4, 5). Nickel sensitivity was tested with 5% nickel sulfate and found to be more common in females compared to males with a female-male ratio of 3.4:1. Around 15.94% of patients showed positive patch test reaction to chlorocresol along with other allergens like parthenium and paraben mix. The ACD to black rubber mix was confirmed in 14.49% of cases whereas 13.04% of patients tested positive for cobalt sulfate and most of them were allergic to nickel also. In our study, 7.24% of patch test was positive for wool alcohol, 5.79% came out to be patch test positive for formaldehyde, and 2.89% were patch test positive for neomycin. Patch test positivity to fragrance mix was found in 5.79% of cases. Despite a few patients presenting to us with clinical features of kumkum dermatitis, none of them showed a positive reaction on patch testing. 

## Discussion

The mean age of the patients in our study was 42.8 years. In a similar study conducted by Davoudi et al. in Iran, 43.6 years was the mean age of the patients [4]. It was found that very young and very old persons are less affected, which could be because people acquire allergic reactions over a period of time and that response gradually diminishes with age. The present study revealed ACD is more common in males than females, which may be explained by the fact that in this part of the country males are more often recruited in industries and construction sites thereby getting more exposure to workplace and environmental allergens. The lesser presentation of females in the study group may be attributed to lower educational status, less awareness about ACD, and utilization of locally available traditional medicaments. The finding is similar to the study conducted by Narendra et al. [5] with male to female ratio of 1.8:1 and the study conducted by Kishore et al. [6] where the ratio was 1.27:1. The higher incidence of ACD in the LSES group in our study can be explained by the fact that manual work in various industries and construction sites involve the people from this group and their lower levels of education in preventing ACD, unavailability of proper protective gear in the workplace, poor personal hygiene, and negligence towards self-care and treatment exposes them to the various allergens causing ACD. We found most of the patients were from urban areas (72%) than rural areas (28%). This is because of the presence of this tertiary care center in an urban area. The place of residence might have affected the pattern of illness, the treatment-seeking behavior, the type of lesion, etc. Statescu et al. showed the distribution of patients from urban areas is 57.38% and from rural is 42.62% [7].

In our study, the most common site of involvement was the hands followed by the head and neck. A similar study conducted by Kashani et al. showed that hand eczema was the predominant presentation of ACD [8]. Whereas in the study by Kasumagic-Halilovic et al., the hand was the commonest site of involvement and the most common allergen was nickel sulfate [9]. We found a significant number of ACD patients with atopy association. In the study conducted by Sharma et al. in Assam, ACD was not rare amongst atopic patients [10].

The higher incidence of ACD to cement in our study might be due to more people being employed in construction work in this part of India and the high percentage of construction workers visiting our OPD. In a study conducted in Mangalore, India, cement was also found to be the most common cause of ACD [11]. With increasing industrialization in India, the construction industry is growing fast and requires a large number of workers leading to an increased incidence of ACD to cement. In our study, we found parthenium was the second most common allergen causing ACD. Airborne contact dermatitis was the most common pattern observed. This pattern was also the most common in the study conducted by Sharma et al. [12].

Para-phenylenediamine is a primary intermediate in permanent hair dyes. In our study, PPD occupied the third place. Allergic contact dermatitis to PPD was commonly noticed in beard areas relatively sparing the scalp. Hsu et al. also described beard dermatitis due to PPD in eight Arabic men [13]. In contrast, Gupta et al. observed dermatitis to PPD more on the scalp and scalp margins [14]. The globalization of the Indian market and easy availability of a variety of hair dyes and colors make it a potential threat for the persons using it.

In general, the most common metal causing sensitization is nickel. In our study, females were found to be more sensitive to nickel as compared to males. This is in accordance with the study done by Nielsen et al. in a group of the Danish population [15]. Nickel can easily cause sensitization as nickel salts are soluble in water and sweat. The most common substances causing nickel sensitization in the study were jewelry such as necklaces due to prolonged contact with skin and metal utensils. The hand was the most common site of involvement in this study and the study conducted by Meding (&) Swanbeck [16]. Chlorocresol is found as a preservative in fungicides, pesticides, and topical corticosteroids. It has been reported by Archer that patients with ACD to nickel and cobalt become sensitive to chlorocresol-containing topical corticosteroid creams [17]. In another study done by Barbaud et al., 5.3% of cases came positive for chlorocresol [18]. 

Our study showed ACD to black rubber mix in 14.49% of cases. In India, a poorer section of people still uses black rubber-mix footwear. In a study done by Chowdhuri et al. in 155 cases, 20% came positive to it on patch testing [19]. Sensitivity to rubber and its constituents was tested with black rubber mix, PPD, and thiuram mix. Rubber was found to be the commonest allergen in footwear. However, the commonest substance to cause allergy in footwear was leather in the study conducted by Chowdhuri et al. [19]. Contact depigmentation in the footwear series was due to rubber. This was also found in studies carried out by Singh et al. [20].

Cobalt sulfate is used as a component in paints for glass and porcelain, jewelry, zippers, buttons, tools, utensils, and instruments. In a study done by Liden et al. [21] in 656 patients, ACD to cobalt was found in 14% of patients which is similar to our finding. Formaldehyde used in the production of urea, textile industry, cosmetics, etc. 5.79% came out to be patch test positive in our study group which can be correlated with the finding of Sharma et al. [22]. Neomycin is a broad-spectrum antibiotic available in topical creams, powders, ointments, and eye and ear drops. In our study, 2.89% were positive for neomycin. The first aid measures delivered by paramedics or the person himself may predispose him or her to neomycin-induced ACD. This finding is also supported by Menezes et al. [23]. Allergic contact dermatitis to fragrance mix is increasing nowadays. A cross-sectional study of five European countries demonstrated the prevalence of fragrance allergy was 1.9% to 2.6% among the general population [24]. We too detected ACD to fragrance mix in some cases. Increased self-look awareness is a major cause of ACD by cosmetics among teenage girls in this region.

Despite a few patients presenting to us with clinical features of kumkum dermatitis, none of them showed a positive reaction on patch testing. Common allergens present in kumkum are Sudan I, aminoazobenzene, Brilliant lake red R, and Cananga oil. In our study, the patch test was done with commercial kumkum. A study done by Nath et al. [25] in the south Indian population showed kumkum as the commonest cause of cosmetic dermatitis evidenced by patch test which might be due to the traditional use of kumkum mixed with turmeric by south Indian women.

## Conclusions

A patch test is an essential tool in diagnosing the etiological agent of ACD. The interpretation of the patch test requires experience and training in considering their relevance and associating the result with the clinical findings of ACD. Many cases of ACD in which etiology could not be found were patch tested and in some cases, allergens were found, which confirmed the diagnosis and were treated accordingly. Upon patch testing, 69% of the patients showed one or more positive patch test results. This depicts that the prevalence of patch test-positive ACD is 69% in this study, which could be because only a standard series was used and the patient may not be sensitized to it. Therefore, the use of a specific additional series (cosmetic, fragrance, industrial) is justified in these cases. When performed and interpreted properly patch test is the only scientific method of investigation and the only definite proof of the state of allergic sensitization. 
